# Low-intensity red and infrared laser effects at high fluences on *Escherichia coli* cultures

**DOI:** 10.1590/1414-431X20154460

**Published:** 2015-07-28

**Authors:** L.L. Barboza, V.M.A. Campos, L.A.G. Magalhães, F. Paoli, A.S. Fonseca

**Affiliations:** 1 Departamento de Biofísica e Biometria, Instituto de Biologia Roberto Alcântara Gomes, Rio de Janeiro, RJ, Brasil; 2 Departamento de Morfologia, Instituto de Ciências Biológicas, Universidade Federal de Juiz de Fora, Juiz de Fora, MG, Brasil; 3 Departamento de Ciências Fisiológicas, Instituto Biomédico, Universidade Federal do Estado do Rio de Janeiro, Rio de Janeiro, RJ, Brasil; 4 Centro de Ciências da Saúde, Centro Universitário Serra dos =rgãos, Teresópolis, RJ, Brasil

**Keywords:** DNA, Escherichia coli, Filamentation, Laser

## Abstract

Semiconductor laser devices are readily available and practical radiation sources providing wavelength tenability and high monochromaticity. Low-intensity red and near-infrared lasers are considered safe for use in clinical applications. However, adverse effects can occur via free radical generation, and the biological effects of these lasers from unusually high fluences or high doses have not yet been evaluated. Here, we evaluated the survival, filamentation induction and morphology of *Escherichia coli* cells deficient in repair of oxidative DNA lesions when exposed to low-intensity red and infrared lasers at unusually high fluences. Cultures of wild-type (AB1157), endonuclease III-deficient (JW1625-1), and endonuclease IV-deficient (JW2146-1) *E. coli*, in exponential and stationary growth phases, were exposed to red and infrared lasers (0, 250, 500, and 1000 J/cm^2^) to evaluate their survival rates, filamentation phenotype induction and cell morphologies. The results showed that low-intensity red and infrared lasers at high fluences are lethal, induce a filamentation phenotype, and alter the morphology of the *E. coli* cells. Low-intensity red and infrared lasers have potential to induce adverse effects on cells, whether used at unusually high fluences, or at high doses. Hence, there is a need to reinforce the importance of accurate dosimetry in therapeutic protocols.

## Introduction

Low-intensity lasers are lightweight, available sources of monochromatic non-ionizing radiation (1). Because they are practical and low-cost, these devices are increasingly being used in health care. In nonphotosynthetisizing cells, laser light absorption occurs via chromophores and alterations in cell physiology have been reported (2). Chromophores, which act as intracellular photoacceptors, are responsible for the biological effects of low-intensity lasers (3). Certain reaction centers in cytochrome c oxidase (Cua and Cub or hemes a and a3) in mammalian cells and cytochrome bd and bo complexes in *Escherichia coli* cells have been described as the main cellular photoacceptors (3). After absorption of laser radiation energy at low fluences by such photoacceptors, transduction processes are responsible for activating intracellular signaling pathways, thereby amplifying the primary photosignal (4). Highly reactive chemical species (i.e., reactive oxygen and nitrogen species) are involved in the transduction processes where they function as second messages, interact with biomolecules, and alter cellular functions and gene expression (4,5). It is possible that photobiological side-effects occur when the antioxidant systems are not capable of protecting the cells against free radical attack. This situation can occur when antioxidant systems are not functioning, or when inadequate exposure to low-intensity lasers at high doses arises. An intracellular imbalance between oxidant and antioxidant contents means that free radicals might occur in cells exposed to low-intensity lasers when high doses are used. At therapeutic doses, sub-lethal DNA damage has been reported after exposure to low-intensity red and infrared lasers in eukaryotic (5 6 7) and prokaryotic cells (8,9).

Although low-intensity laser radiation can potentially damage DNA, therapeutic protocols based on it are used successfully to improve wound healing (10), accelerate the repair of skin, cartilage and bone, to treat nerve injuries and relieve inflammation (11) and pain (12). The scientific basis of laser applications in therapy is the so-called biostimulation (or biomodulation) effect, which results from alterations of intracellular processes, mainly via an increase in metabolism and the rate of cell division (2).

The biological effects of low-intensity lasers are dependent on the exposure parameters used. Energy densities, directionality, high monochromaticity and emission mode properties are characteristics that enable semiconductor laser devices to treat various diseases, and the different clinical protocols suggested for their use can be found in specialized literature on this topic (11) and in guides on laser devices. These protocols are based on low-energy densities (fluences) or low-power densities and for this reason low-intensity lasers are considered safe for clinical applications. Also, red and near-infrared radiation (600 up to 1300 nm) is not considered to induce significant adverse effects in biological tissues (2), unlike ultraviolet radiation, which induces hyperpigmentation, aging and carcinogenesis (13). Under low fluences (0.1 up to 100 J/cm^2^), low-intensity lasers are considered to generate nonthermal and nondestructive effects (1). However, high energy densities and intensities are deposited in a small volume and over a short time period, thereby delivering high-dose radiation to the biological tissue exposed to such lasers. Hence, the clinical outcomes of laser use depend on delivery of accurate doses of laser radiation and ensuring that adverse effects cannot occur through accidental high-dose exposure. However, few experimental studies on the biological effects induced by low-intensity lasers at unusual doses exist, making research in this area important as undesirable effects from low-dose lasers can occur via accidental exposure or when non-calibrated devices are used. Therefore, the work presented here investigated the survival, filamentation induction and morphology of *E. coli* cells deficient in repair of oxidative DNA lesions when exposed to low-intensity red and infrared laser radiation at unusually high fluences.

## Material and Methods

### Low-intensity red and near-infrared lasers

Therapeutic low-intensity red and near-infrared lasers (Photon Lase III) were purchased from DMC Equipamentos Ltda. (Brazil). The laser parameters are shown in Table 1.

**Table:**
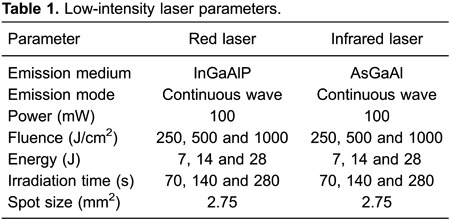

### *E. coli* cell survival

Cultures of *E. coli* AB1157 (wild-type), JW1625-1 (deficient in endonuclease III) and JW2146-1 (deficient in endonuclease IV) were exposed to low-intensity red and infrared lasers and their survival rates were evaluated. From stocks in stationary growth phase, cultures of these strains were prepared to attain their exponential growth phase (i.e., 10^8^ cells/mL; 2–3 h, 37°C). Other experiments were carried out with cultures of the same *E. coli* strains in the stationary growth phase (10^10^cells/mL; 18 h, 37°C). Bacterial cells were centrifuged twice (700 *g*, 15 min) and resuspended in saline (0.9% NaCl) each time. Aliquots (50 µL, n=5, for each fluence) of the bacterial suspensions (10^8^ cells/mL) were exposed, at room temperature and under white light (fluorescent lamps), to low-intensity red and infrared lasers. The exposure time of the cells was automatically adjusted by the laser device as a function of the fluence. The laser device was positioned such that almost all the surface of the bacterial aliquot suspension was covered by the laser beam. Controls were bacterial suspensions not exposed to lasers. Immediately after exposure to a laser, the bacterial suspensions were diluted in normal saline and spread onto Petri dishes containing solidified rich medium (1.5% agar). Bacterial colonies were counted after incubation (37°C, 18 h) and the survival fractions were calculated (14).

### Bacterial filamentation assays

To evaluate filamentation induction, exponential and stationary *E. coli* AB1157, JW1625-1, and JW2146-1 cultures were obtained and exposed to low-intensity red and infrared lasers as described in the bacterial survival assay. Bacterial suspensions not exposed to lasers were used as controls. Immediately after exposure, aliquots (20 µL) were withdrawn, spread onto microscopic slides and stained by the Gram method (15). Bacterial cells were visualized using a Carl Zeiss Axio Scope A1 microscope (Germany) equipped with an A-plan 40/0.65 objective, a 0.90 condenser and a 100W halogen lamp. The images were captured with an AxioCam HRc Sony 12M color microscopy camera (Carl Zeiss), using AxioVision software. Thereafter, the images were analyzed by Image-Pro Plus 6.0 software for Windows XP (Media Cybernetics, Inc., USA) to determine the bacterial filamentation percentages. A bacterial filament was considered to be 2.5 times the average area of a bacterial cell. Experiments were carried out in duplicate and the results represent the mean of three independent assays.

### Bacterial morphological measurements

Bacterial suspensions of *E. coli* AB1157, JW2146-1, and JW1625-1 (10^8^ cells/mL) were exposed to low-intensity red and infrared lasers as described above in the bacterial survival and filamentation assay methods. Immediately after laser exposure, aliquots were spread onto microscopic slides and stained by the Gram method (15). Bacterial cells were visualized by light microscopy (300 cells for each laser exposure), as described in the bacterial filamentation assay method.

### Statistical analysis

Data are reported as means±SD of the bacterial survival fractions, the bacterial filament percentages, and the surface area of the bacterial cells. One-way analysis of variance (ANOVA) was performed to verify potential statistical differences, followed by the Tukey post-test with P<0.05 indicating statistical significance. InStat software for Windows XP (GraphPad Software, USA) was used to perform the statistical analyses.

## Results

### Survival of *E. coli* cultures exposed to low-intensity red and infrared lasers

The survival fractions of exponentially grown *E. coli* AB1157, JW1625-1 and JW2146-1 cultures exposed to low-intensity red and infrared lasers are reported in Table 2. The data in this table show that exposure to these lasers did not significantly alter the survival fractions of the *E. coli* AB1157 and JW1625-1 cultures. However, red and infrared lasers significantly (P<0.05) decreased the survival fractions of JW2146-1 at the higher fluence (1000 J/cm^2^) evaluated herein.

**Table:**
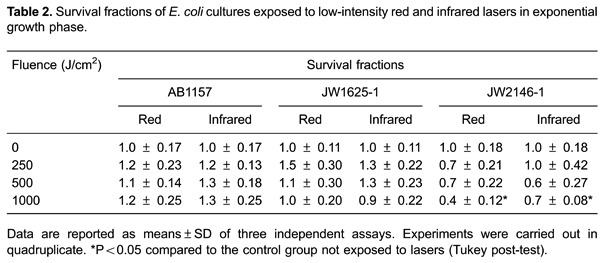

The survival rates of stationary cultures of the same *E. coli*strains were evaluated to verify whether the low-intensity red and infrared laser effects are dependent on the physiological conditions of the cells (Table 3). Stationary *E. coli* AB1157 cultures had survival fractions similar to those of the exponential cultures. However, *E. coli* JW1625-1 had an increased survival fraction after exposure to red laser at the higher fluence level. No significant alteration of the survival fraction was obtained for *E. coli* JW1625-1 after infrared laser exposure. In contrast to the decreased survival fractions of the exponential cultures of *E. coli* JW2146-1, the survival fractions of stationary JW2146-1 cultures were not significantly modified by exposure to low-intensity red and infrared lasers.

**Table:**
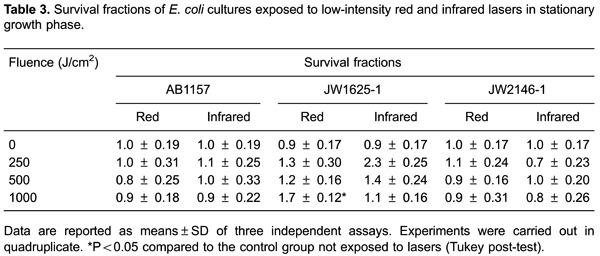

### Filamentation induction in *E. coli* cultures exposed to low-intensity red and infrared lasers

**Figure 1:**
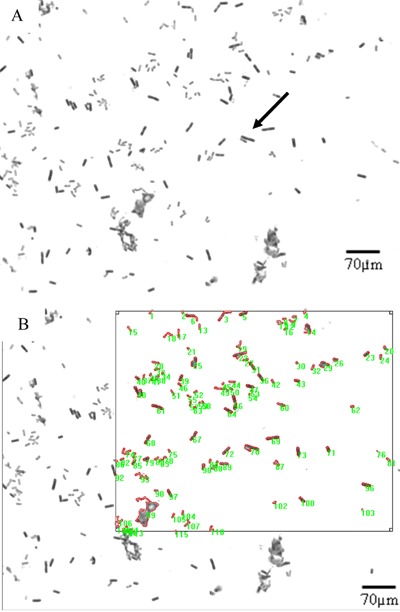
Representative images of bacterial filamentation from AB1157 cultures in the stationary growth phase. *A*, Arrow denotes bacterial filamentation; *B*, same image illustrating how the image analysis was performed. A bacterial filament was considered to be present in a bacterium when the area of the bacterial cell was 2.5-times larger than the mean value of the area.

Figure 1 shows a photograph of representative cells from *E. coli* AB1157 cultures during the exponential growth phase (1A). Figure 1Bshows the bacterial cell image analysis. The bacterial filament percentages in exponential phase *E. coli* cultures are shown in Table 4. Data in this table show that the red and infrared lasers did not significantly induce the filamentation phenotype in *E. coli* AB1157. Also, infrared laser treatment did not significantly induce filament formation in *E. coli* JW1625-1 and JW2146-1 cultures. However, in the JW1625-1 cultures, exposure to low-intensity red laser significantly (P<0.05) induced an increase in the percentage of bacterial filaments, but in JW2146-1 cultures this effect was significant only at mid fluence (500 J/cm^2^).

**Table:**
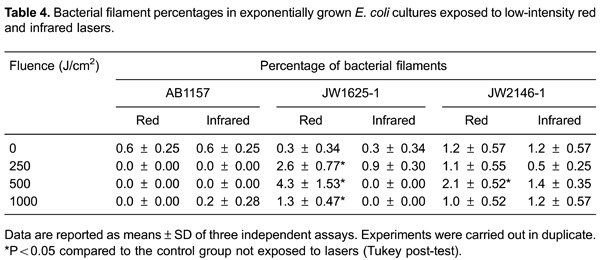

Stationary *E. coli* cultures were also exposed to red and infrared lasers to evaluate filamentation induction (Table 5). Similar to the results observed with the exponential cultures, *E. coli* AB1157 exposure to red laser treatment did not induce significant filamentation, but exposure to infrared laser at the higher fluence (1000 J/cm^2^) increased the level of this phenotype. In contrast to the results of the exponential cultures, red laser exposure did not induce significant filamentation in stationary *E. coli* JW1625-1. Interestingly, laser exposure significantly (P<0.05) reduced the filament percentage in stationary *E. coli* JW2146-1, except at 500 J/cm^2^ (no significant alteration) and at 1000 J/cm^2^ where a significant (P<0.05) increase in bacterial filaments was seen.

**Table:**
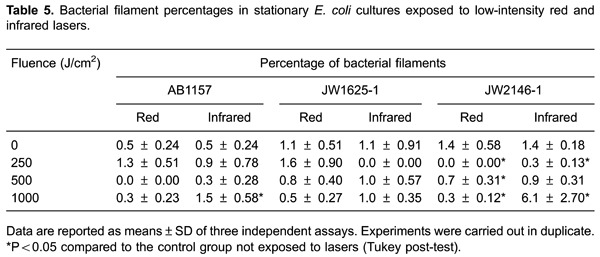

### Effect of low-intensity red and infrared lasers on the surface area of *E. coli* cells

The surface area of individual *E. coli* cells was evaluated after exposure to lasers at high fluences (Tables 6 and 7). The data in Table 6 show that exposure to red and infrared lasers significantly (P<0.05) increased the surface area of exponential *E. coli* AB1157 cells. However, exposure to the red laser did not induce significant alteration of the surface area of *E. coli* JW1625-1 cells; infrared laser exposure at the highest fluences (500 and 1000 J/cm^2^), however, did reduce the surface area of these cells. No significant alterations to the surface area of *E. coli* JW2146-1 exposed to red and infrared lasers were observed.

**Table:**
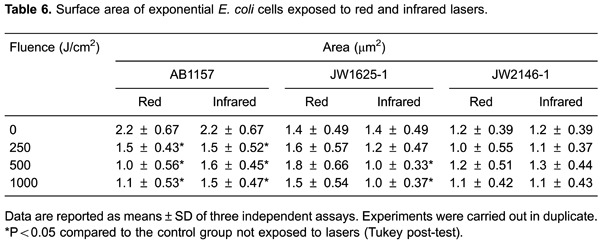

**Table:**
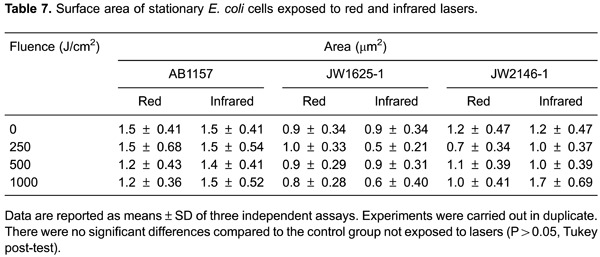

To verify whether stationary cells, which differ physiologically to exponential cells, could be altered by laser exposure, the surface area of these cells was also evaluated. The data in Table 7 show that low-intensity red and infrared lasers did not induce significant alterations to the surface areas of stationary *E. coli* AB1157, JW1625-1, and JW2146-1 cells.

## Discussion

Some clinical protocols based on low-intensity red and infrared lasers were proposed by empirical professional practice. Also, current device guides do not contain recommendations that prevent medical professionals from delivering high radiation doses to patients during laser exposure. Despite some discrepancies, experimental studies have mostly described the positive effects induced by such lasers when used at therapeutic doses. However, the effects of accidental exposure to high-dose radiation or exposure to non-calibrated laser devices have been neglected. Our research shows that, at high fluences, low-intensity red and infrared laser radiation used to treat soft tissue diseases can induce lethal effects on *E. coli* cultures, depending on the DNA repair mechanisms of the strain and the physiological condition of the cells (i.e., stationary or exponentially growing cells) (Tables 2 and 3). In previous studies, we have shown that *E. coli* (AB1157) cells proficient in DNA repair mechanisms are resistant to red and infrared laser exposure at fluences or doses in the therapeutic range (16 17 18). The data in Tables 2 and 3 suggest that these cells are also not inactivated by red and infrared laser exposure at high fluences. However, high fluences of red and infrared lasers decrease the survival rates of exponential phase *E. coli* cultures deficient in endonuclease III (JW1625-1) and *E. coli* cultures deficient in endonuclease IV (JW2146-1) (Table 2). As part of its base excision repair mechanism, endonuclease III repairs apurinic/apyrimidinic sites and damaged pyrimidines (19,20). Similarly, endonuclease IV acts on apurinic/apyrimidinic sites and oxidatively damaged bases in *E. coli* (21 22 23 24). Data obtained in our study indicated that sub-lethal oxidative lesions in DNA are induced in cells exposed to low-intensity red and infrared lasers, and that the survival of cells with failing DNA repair mechanisms decreased when exposed to such radiation. However, stationary endonuclease IV-deficient cells are not sensitive to lasers and the viability of endonuclease III-deficient cells is increased by red laser exposure at the higher fluence level we evaluated (1000 J/cm^2^). These results suggest that both endonuclease III- and endonuclease IV-deficient *E. coli* cells respond to low-intensity lasers depending on their physiological condition. Nevertheless, laser-induced effects on endonuclease III-deficient cells at high fluence might be related to an increase or acceleration of cellular proliferation (biostimulation or biomodulation effect) (1,17) despite this effect not being observed at the similar fluences used in this study.

Low-intensity red and infrared lasers at unusually high fluences did not induce filamentation in exponential phase wild-type *E. coli* AB1157 cultures (Table 4). Also, endonuclease III and endonuclease IV-deficient *E. coli* cultures did not present this phenotype when exposed to an infrared laser. However, at therapeutic fluences, low-intensity lasers induce filamentation in cultures of these bacterial strains (17,18,25,26). At high laser fluences, the bacterial cells could use other defense mechanisms against laser radiation because bacterial survival was not affected, except for *E. coli* JW2146-1 cultures at 1000 J/cm^2^. Then again, the data obtained with endonuclease III and endonuclease IV at mid laser fluence (500 J/cm^2^) agree with these previous data. To confirm whether physiological conditions can influence the effects of low-intensity lasers on cells, a filamentation assay was also performed with stationary *E. coli* cultures (Table 5). Except at 1000 J/cm^2^, exposure to lasers did not induce filamentation in wild-type and endonuclease III-deficient *E. coli*cultures in the stationary growth phase. Also, red and infrared lasers at high fluences induced different effects on the filamentation phenotype in endonuclease IV-deficient *E. coli* cultures, except at 1000 J/cm^2^. In fact, these lasers induced the filamentation phenotype at therapeutic fluences in stationary endonuclease IV-deficient cells (16,18). These data suggest that, at unusually high laser fluences, bacterial cells could use other defense mechanisms (antioxidant mechanisms) different from those used at therapeutic fluences.

Use of the filamentation assay has permitted evaluation of the induction of this phenotype as indicative of DNA damage by low-intensity laser at therapeutic fluences (16 17 18). However, cells exposed to lasers can present other morphological changes and surface area measurements were carried out in wild-type, endonuclease III-deficient (JW1625-1) and endonuclease IV-deficient (JW2146-1) *E. coli* cells. Indeed, the data in Table 6 show that exposure to low-intensity red and infrared lasers decreased the surface areas of exponential phase wild-type *E. coli* cells. Also, the surface areas of exponential *E. coli* JW1625-1 cells decreased when exposed to infrared laser at the highest fluences (500 and 1000 J/cm^2^) but not by red laser exposure. Exposure to red and infrared lasers did not alter the surface areas of *E. coli* JW2146-1 cells at exponential phase. In stationary growth phase, the low-intensity red and infrared lasers did not modify the surface areas of wild-type *E. coli* AB1157, JW1625-1 and JW2146-1 cells (Table 7). Some authors have reported that low-intensity lasers alter the function of ion channels in the plasmatic membrane (27,28) and in the mitochondrial membrane (29). The results of our morphological analyses can be explained by the effects of the low-intensity lasers on such membrane ion channels. However, additional studies are necessary to evaluate whether such lasers, by direct or indirect mechanisms, affect the functions of membrane ion channels in bacterial cells.

However, despite our results suggesting that free radicals are involved in the laser-induced effects on cell viability and morphology of the bacterial cells, it is possible that the transient thermal effects of the low-intensity lasers (1) are involved in the biological effects reported in this work.

In conclusion, the data from this study show that high fluences of low-intensity red and infrared lasers are lethal, induce a filamentation phenotype, and alter the morphology of *E. coli* cells. Low-intensity red and infrared lasers affect bacterial cells whether used at unusually high fluences or high doses, and our findings reinforce the need for accurate dosimetry in therapeutic protocols.
